# Supercritical Carbon Dioxide and Microwave-Assisted Extraction of Functional Lipophilic Compounds from *Arthrospira platensis*

**DOI:** 10.3390/ijms17050658

**Published:** 2016-05-05

**Authors:** Diego A. Esquivel-Hernández, Víctor H. López, José Rodríguez-Rodríguez, Gibrán S. Alemán-Nava, Sara P. Cuéllar-Bermúdez, Magdalena Rostro-Alanis, Roberto Parra-Saldívar

**Affiliations:** 1 Escuela de Ingenieria y Ciencias, Tecnologico de Monterrey, Campus Monterrey, Ave. Eugenio Garza Sada 2501, Monterrey, N.L. 64849, Mexico; A00796778@itesm.mx (D.A.E.-H.); jrr@itesm.mx (J.R.-R.); gibran.aleman@itesm.mx (G.S.A.-N.); sara.cuellar@itesm.mx (S.P.C.-B.); magda.rostro@itesm.mx (M.R.-A.); 2 Tecnologia Ambiental Biomex S.A. de C.V., Volcan Jorullo 5268, Zapopan, Jalisco 45070, Mexico; victor.biomex@hotmail.com

**Keywords:** supercritical fluid extraction, microwave assisted extraction, *Arthrospira platensis*, cyanobacteria, functional lipophilic compounds, β-carotene, α-tocopherol, γ-linolenic acid

## Abstract

*Arthrospira platensis* biomass was used in order to obtain functional lipophilic compounds through green extraction technologies such as supercritical carbon dioxide fluid extraction (SFE) and microwave-assisted extraction (MAE). The temperature (T) factor was evaluated for MAE, while for SFE, pressure (P), temperature (T), and co-solvent (ethanol) (CS) were evaluated. The maximum extraction yield of the obtained oleoresin was (4.07% ± 0.14%) and (4.27% ± 0.10%) for SFE and MAE, respectively. Extracts were characterized by gas chromatography mass spectrometry (GC-MS) and gas chromatography flame ionization detector (GC-FID). The maximum contents of functional lipophilic compounds in the SFE and MAE extracts were: for carotenoids 283 ± 0.10 μg/g and 629 ± 0.13 μg/g, respectively; for tocopherols 5.01 ± 0.05 μg/g and 2.46 ± 0.09 μg/g, respectively; and for fatty acids 34.76 ± 0.08 mg/g and 15.88 ± 0.06 mg/g, respectively. In conclusion, the SFE process at P 450 bar, T 60 °C and CS 53.33% of CO_2_ produced the highest yield of tocopherols, carotenoids and fatty acids. The MAE process at 400 W and 50 °C gives the best extracts in terms of tocopherols and carotenoids. For yield and fatty acids, the MAE process at 400 W and 70 °C produced the highest values. Both SFE and MAE showed to be suitable green extraction technologies for obtaining functional lipophilic compounds from *Arthrospira platensis*.

## 1. Introduction

Microalgae and cyanobacteria cultivation has attracted the interest of industrial companies and the scientific community as a natural and sustainable biomass source for bioactive compounds [1,2]. Furthermore, they are capable of fixating CO_2_ from the atmosphere, they do not need to use agricultural lands to grow, they are fast growers and they have a high tolerance to varying environmental conditions [3,4]. *Arthrospira platensis* is a photosynthetic filamentous microorganism [5] considered a “super food” due to its pharmaceutical and nutraceutical properties [6], including antiproliferative, antitumor, antifungal, antibacterial, antimalarial, antiviral, and antimycotic characteristics [7,8]. Commercially available products of *A. platensis* contain approximately 65% protein, 20% f carbohydrate, 7% minerals, 5% lipids and 8% moisture [9]. Lipids are important cellular constituents that have multiple critical roles in cellular functions [10], besides their multiple applications as nutraceuticals, food additives [11], antimicrobials [12], and biofuels [13]. The main components of the algae lipid fraction are fatty acids (FA), phospholipids, tocopherols, waxes, sterols, hydrocarbons, ketones and pigments (carotenoids, chlorophylls) [14]. Traditional extraction techniques of oils from algae are based on the same technology of plant oil extraction (organic solvent extraction, Soxhlet). These techniques require high energy and are time-consuming, and they also polluting the environment with hazardous solvents. Therefore, sustainable technologies must be implemented, such as supercritical fluid extraction (SFE) and microwave-assisted extraction (MAE). Both use eco-friendly solvents, and require lower energy input while reducing extraction time [15]. In the case of SFE, one of its advantages is the use of carbon dioxide (CO_2_) as the main solvent for the extraction, since it has a low critical point (T 30.9 °C and P 73.9 bar). Also, it is cheap, environmentally friendly and generally recognized as safe (GRAS) by the Food and Drug Administration (FDA) [16]. Furthermore, CO_2_ has a high diffusivity with a tunable solvent strength [17]. On the other hand, MAE uses non-ionizing electromagnetic waves, enabling the extraction of bioactive compounds from complex biological matrices. It also requires less solvent volume and time for the extraction [18,19]. Additionally, MAE offers more effective and selective transfer heating (without heat transfers), allowing the reduction of thermal gradients, and can exploit the use of different types of solvents to increase the range of polarity of the extraction, thus increasing the variety and amount of metabolites extracted [20,21]. SFE with CO_2_ has been previously used for the extraction of functional lipophilic compounds from *Arthrospira platensis* such as carotenoids [22,23], fatty acids [24,25] and tocopherols [26,27]. However, all of these studies have been performed for individual compounds and did not consider the interaction between the lipophilic compounds and process parameters. On the other hand, for MAE technology, the available information about *Arthrospira* only covers the extraction of protein pigments (C-phycocyanin) [28], while for other microalgae/cyanobacteria, the information is scarce and, similarly, only focused on pigments [21,29] without evaluation of temperature in the process. To our knowledge, there are no reports on the extraction of functional lipophilic compounds from *Arthrospira* by MAE.

The data provided below demonstrate the advantages of using green extraction technologies, since they are environmentally friendly and faster than traditional solvent extraction. Therefore, the goal of this study was to explore the effect of the extraction factors on the content of the functional lipophilic compounds in the extracts obtained by SFE and MAE, in order to determine the most important extraction factors and provide a framework for future work. In the present study, the $2^{k}$ model was chosen, as it allows the analysis of a variety of factors with a minimum of experimental runs [30,31]. The factors considered for this study were pressure, temperature and co-solvent (ethanol) for SFE and temperature for MAE. 

## 2. Results and Discussion

### 2.1. Effect of Conditions of Supercritical Fluid Extraction (SFE) and Microwave-Assisted Extraction (MAE) on Extraction Yield in Arthrospira platensis Extracts

SFE and MAE conditions were tested to cover a wide range of lipophilic compounds. Table 1 lists the different experiments performed (the first eight experiments correspond to SFE (*n* = 2) and the last two experiments correspond to MAE (*n* = 3)) along with the extraction yields (as % *w*/*w*) showed as the mean ± standard deviation. For SFE, the highest extraction yield (4.07% ± 0.14% *w*/*w*) was achieved with experiment BDF, at 450 bar, 60 °C and 53.33% of co-solvent. When working with CO_2_ at supercritical conditions, the yield increases with the addition of the co-solvent, meaning that the extracted compounds are of different polarity and, therefore, the process becomes less selective [32]. For yield extractions by SFE, the statistical analysis (ANOVA) showed that the temperature and co-solvent were significant factors (*p <* 0.05), where the highest temperature and highest amount of co-solvent gave the highest yield. In this response, pressure was not a significant factor (*p >* 0.05). For SFE, it has been reported that the main effect of a co-solvent is the solubility enhancement that results from an increase in solvent density and/or intermolecular interactions between the co-solvent and the solute [33]. According to our results, the highest yield was six times above the reported value for *Arthrospira platensis* extracts by SFE-CO_2_ [23], which can be attributed to the absence of co-solvent in the previous studies. In the case of MAE, the highest temperature (experiment H, 70 °C) produced the highest yield (4.27 ± 0.10) (*p <* 0.05). Therefore, this shows that the use of higher temperatures in SFE and MAE led to the highest yields in these extraction processes. Moreover, the highest yield in MAE can also be attributed to the solvents used, as they have been widely reported as effective for obtaining lipophilic compounds with different polarities from different microalgae/cyanobacteria sources [34,35].

### 2.2. Effect of Conditions of SFE and MAE on Carotenoids Content in Arthrospira platensis Extracts

**Figure 1:**
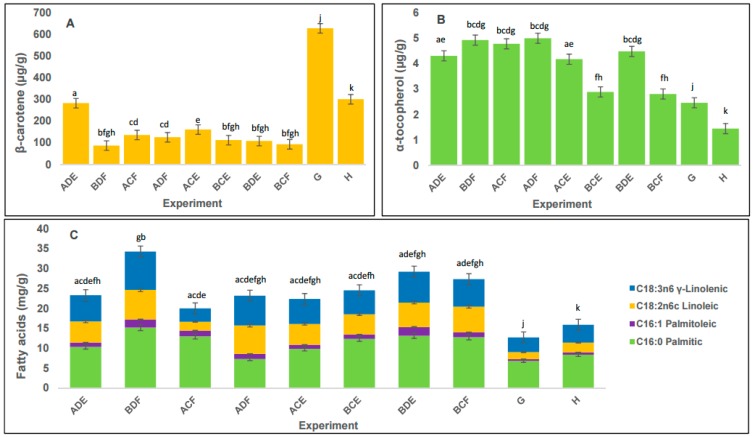
Functional lipophilic compound concentration of SFE and MAE extracts of *Arthrospira platensis*. (**A**) β-Carotene (μg/g); (**B**) α-tocopherol (μg/g); (**C**) fatty acids (mg/g). Values are presented as a mean ± standard deviation (*n* = 2 for SFE and *n* = 3 for MAE). Different letters in the bars are significantly different (LSD test, *p <* 0.05).

Figure 1A shows the values (means) of the concentration of carotenoids obtained for all experiments corresponding to SFE and MAE. For SFE, the highest content of carotenoids (283 ± 0.10 μg/g) was achieved (ADE) at 150 bar, 60 °C and 26.70% of co-solvent. Extraction with this pressure resulted in the highest efficiency in terms of carotenoid content; however, we observed that selectivity decreases at 450 bar (high pressure), because the experiment (BDF) that gave the highest yield uses different pressure and co-solvent levels compared with the experiment (ADE) which produced the highest content of carotenoids. This means that we extracted more compounds other than carotenoids, and these lipophilic compounds suffer a dilution effect. For the carotenoid content, the statistical analysis (ANOVA) showed that pressure was a significant factor (*p <* 0.05), while the temperature and co-solvent were not significant factors (*p >* 0.05). These results are similar to those obtained previously for *Arthrospira* species [22,36,37]. In the case of MAE, the highest content of carotenoids was obtained at experiment G, 50 °C (629 ± 0.13 μg/g) (*p <* 0.05). Furthermore, it was observed that a higher temperature (experiment H, 70 °C) produced the lowest content. These results agree with various reports, stating that the most sensitive carotenoids (violaxanthin) in MAE usually start degrading at temperatures higher than 60 °C [21]. Zhao *et al.* [29] reported excellent results with the solvents used in the MAE extraction of astaxanthin from *Haematococcus pluvialis*, where a high content of carotenoids was obtained. Also, our results agree with previous reports that suggest that efficient and fast extraction of carotenoids can be obtained using MAE without high pressure. Furthermore, the outstanding performance of MAE for carotenoids extraction could be attributed to the wall composition of *A. platensis,* since previous studies of pigment extractions report low efficiency of MAE with microalgae/cyanobacteria species that have a thick exopolysaccharide envelope [21]. Our results, in accordance with other reports for carotenoids extraction with SFE [38,39], show that the use of temperatures at about 60 °C led to the highest content of carotenoids from *A. platensis*. In the case of MAE, the highest content was observed at 50 °C.

### 2.3. Effect of Conditions of SFE and MAE on Tocopherols Content in Arthrospira platensis Extracts

Figure 1B shows the concentration (means) of tocopherols obtained by all experiments of SFE and MAE. According to the results from the mass spectrometer (Supplementary Materials Figure S1 and Table S1), the only element of tocopherols found in the samples was α-tocopherol. For SFE, the highest content of α-tocopherol was 5.01 ± 0.05 μg/g with experiment ADF (150 bar, 60 °C and 53.33% of co-solvent). Temperature was a significant factor according to the ANOVA analysis (*p <* 0.05). The highest content of tocopherols was lower than previously reported [27]; this may be due to the use of ethanol as a co-solvent in this study. Other studies have reported the use of non-polar solvents for tocopherol extractions [40]. Moreover, the main difference may be due to physiological differences between strains of *Arthrospira* used in previous reports and in this study [41]. For MAE, the highest content of tocopherols was with experiment G, 50 °C (2.46 ± 0.09 μg/g) (*p <* 0.05), where the higher temperature negatively affected the content of tocopherols. This can be attributed to a possible degradation by heat [42]. Comparing the content of α-tocopherol in the extracts obtained with MAE, it was lower than the content obtained by SFE. Therefore, in this special case, the polarity obtained with SFE was better for these metabolites.

### 2.4. Effect of Conditions of SFE and MAE on Fatty Acids Content in Arthrospira platensis Extracts

Fatty acids represent one of the major building blocks of lipids [43]. They can be used in different applications such as biofuels, food, *etc.*; therefore, it is important to determine the fatty acid content in the extracts obtained with SFE and MAE. Figure 1C shows the concentration (means) of fatty acids obtained for all experiments corresponding to SFE and MAE. For SFE, the highest content of fatty acids (34.76 ± 0.08 mg/g) was obtained with experiment BDF, with 450 bar, 60 °C, and 53.33% of co-solvent. Extraction with high pressure, high temperature and high co-solvent level gives the highest proficiency in terms of fatty acids extraction. Moreover, pressure, temperature and co-solvent were all significant factors (*p <* 0.05). The highest fatty acid content was found in treatments with 450 bar experiments (BDF, BCE, BDE and BCF); as a consequence, pressure was an important parameter in the process of extraction of fatty acids by SFE. These results agree with previous reports of extraction of fatty acids with SFE. Machmudah *et al.* [44] reported that an increase in the extraction pressure generates an increase in the density of carbon dioxide and, consequently, an increment in the solvating power for fatty acids. In the case of temperature, the results agree with those previously reported for SFE [45,46,47]. An increase in temperature at 450 bar resulted in a higher content of fatty acids, while an increase in temperature at 150 bar resulted in a lower fatty acid content. These results are explained by the “crossover point”: this parameter depends on analyte-supercritical fluid interactions, and appears when the pressure of a supercritical fluid is constant while the temperature increases. For example, if this pressure is below the “crossover point”, an increase in temperature leads to lower solvent strength of the fluid due to the decrease in fluid density. Above the “crossover point”, an increase in temperature can improve the extraction efficiency despite the decrease in fluid density, since the vapor pressure of the analyte is increased [46]. In the case of the co-solvent (ethanol), it acts as an important factor in SFE for the enhancement of the solubility of lipophilic compounds (fatty acids, β-carotene, squalene among others) [33]. For fatty acids, a significant increase in the solubility has been reported when ethanol is used as a co-solvent, due to the interactions of hydrogen bonds [33]. The composition of the fatty acids fraction in SFE extracts was: palmitic acid—45.10%, palmitoleic acid—4.91%, linoleic acid—21.27% and γ-linoleic acid—27.71%, agreeing partially with previous reports [25,48]. For example, for palmitic acid in *Arthrospira*, Sajilata *et al.* [25] reported a higher content (53.09%), while Andrich *et al.* [48] reported a lower content (39.10%). These differences may be due to the effect of growth conditions of cyanobacteria on the composition of their metabolites [49].

For MAE the highest content of fatty acids (15.88 ± 0.06 mg/g) (*p* < 0.05) was obtained with experiment H, 70 °C. This result is similar to those obtained by extraction yield at the same temperature. In the case of MAE, temperature is an important factor, since higher temperature produces the highest yield of compounds, although with the limitation of the thermal stability of the compounds present in the extract. According to the obtained results, the content of tocopherols and carotenoids decreased with increasing temperature. The composition of fatty acids in MAE extracts was found to be palmitic acid—52.64%, palmitoleic acid—3.72%, linoleic acid—15.47% and γ-linoleic acid—28.15%. Moreover, the content of γ-linolenic acid from MAE extracts was similar to the content of γ-linolenic acid from SFE extracts. However, there were differences in the content of palmitic acid, where MAE extracts (52.64%) were higher than SFE extracts (45.10%). In respect to linoleic acid, SFE extracts had higher content (21.27%) than MAE extracts (15.47%). This was due to differences in the affinity of the solvents used in the extraction process. This information is important since MAE offers higher purity extracts than other extraction technologies [28], so this can be exploited according to the different applications of fatty acids. For instance, palmitic acid can be used for biofuels production [50,51], while γ-linolenic acid has shown positive effects in treating inflammatory conditions [52,53,54]. Therefore, these findings provide information for the production of functional lipophilic compounds such as palmitic and γ-linolenic acid from a natural and sustainable source.

## 3. Materials and Methods

### 3.1. Samples and Chemicals

Cyanobacteria samples (*Arthrospira platensis*) were obtained from Tecnologia Ambiental Biomex S.A. de C.V. [55] (Guadalajara, Mexico). *Arthrospira* was grown in open raceway ponds with modified Jourdan medium [56] during 45 days. The geographical location of the ponds was 20°14′0″ N, 103°35′0″ W. The biomass was harvested with a mesh, air dried to 20% moisture in order to maintain the stability of lipophilic compounds and stored under dry and dark conditions until the extracts were prepared (2 weeks). Methanol, ethyl acetate and light petroleum 60–80 °C boiling point were purchased from Tedia (Monterrey, Mexico). Fatty acids methyl esters, β-carotene, undecanoic acid, sulfuric acid and α-tocopherol were obtained from Sigma-Aldrich (Toluca, Mexico). Carbon dioxide (Industrial grade) was acquired from Praxair S.A. (Guadalajara, Mexico) and ethanol from Reactivos Guadalajara (Guadalajara, Mexico).

### 3.2. Supercritical Fluid Extraction (SFE)

All extractions were carried out in a pilot-scale plant for supercritical fluid extraction (Biomex, Mexico) with a 100 mL extraction vessel (Waters Thar SFC SFE 100 Equipment). All extractions were carried out using a flow of 15 g/min of CO_2_ for 50 min and ethanol/water 96% (*v*/*v*) was used as co-solvent. An automated back pressure regulator (ABPR) controlled the extraction pressure, carbon dioxide pump was purchased from Thar Instruments (Pittsburgh, PA, USA) and the co-solvent pump from Waters (Milford, CT, USA).

For each experiment, the extraction vessel was filled with 20 g of cyanobacteria powder (milled with mortar and pestle). The solvent/feed ratio (*g*/*g*) was 37.5 in order to obtain the highest possible yield for SFE [57]. Pressure and temperature in the separator were the same in all experiments (1 bar and 25 °C). The effects of three factors, pressure (P), temperature (T) and co-solvent (CS) on the extraction of functional lipophilic compounds were examined using a $2^{k}$ (150 (A), 450 (B)); T (°C) (45 (C), 60 (D)); CS (as % of CO_2_) (26.7 (E), 53.33 (F)). All extracts were concentrated in a vacuum rotary evaporator IKA Works (Wilmington, NC, USA) and kept under N_2_ at −20 °C in the dark. The selected response variables were extraction yield, concentration of carotenoids, concentration of tocopherols and concentration of fatty acids. The statistical method used for the data analysis was ANOVA, using the statistical package Minitab version 16 (State College, PA, USA). All extractions were performed in duplicate.

### 3.3. Microwave-Assisted Extraction (MAE)

All extractions were carried out in a microwave-assisted extraction equipment MARS 5^®^ (CEM Corporation, Matthews, USA) with a 100 mL extraction vessel GreenChem™. The extraction vessel was made of PFA Teflon™. All extractions were done using methanol/ethyl acetate/light petroleum (1:1:1 *v*/*v*) at 400 W power, 1 bar, and 15 min extraction time. For each experiment, the extraction vessel was filled with a 0.06 ratio of biomass/solvent (*w*/*v*). The effect of temperature was tested at two experimental levels T (°C) (50 (G), 70 (H)). All extracts were centrifuged at 4500 rpm, 4 °C for 7 min; the supernatants were transferred to evaporation flasks for concentration in a vacuum rotary evaporator (IKA Works, Wilmington, NC, USA) and kept under N_2_ at −20 °C in the dark; the residual pellets were discarded. The selected response variables were extraction yield, concentration of carotenoids, concentration of tocopherols and concentration of fatty acids. The statistical method used for the data analysis was ANOVA, using the statistical package Minitab version 16 (State College, USA). All Extractions were performed in triplicate.

### 3.4. Carotenoids Analysis

All extracts were made up to a final volume of 15 and 5 mL with ethyl acetate for SFE and MAE extracts, respectively. Total carotenoid content was estimated with UV-Vis spectrophotometer Hach DR5000 (Loveland, CO, USA) at absorbance maxima of 450 nm for β-carotene. Carotenoid content was calculated using an $E_{cm}^{\%}$ of 2100 and was quantified as equivalents of β-carotene in mg/g.

### 3.5. Tocopherols Analysis

Tocopherols were analyzed with an Agilent 6890N GC (Agilent Technologies, Santa Clara, CA, USA) equipped with a HP-5MS capillary column (30 m, 0.25 mm i.d., 0.25 μm film thickness) and a mass spectrometer 5973 N as a detector. The carrier gas was helium at a flow rate of 0.8 mL/min. The column temperature was kept initially at 190 °C for 1 min, then gradually increased to 300 °C at 15 °C/min, and finally, maintained at 300 °C for 10 min. For GC-MS detection, an electron ionization system was used with 70 eV of energy. An aliquot of the extracts (1 µL) was injected automatically with 20:1 in split mode. Injector and detector temperatures were set at 270 and 230 °C, respectively.

### 3.6. Fatty Acids Analysis

Fatty acids were determined through derivatization to fatty acid methyl esters (FAMEs). To prepare FAMEs of the extracts, samples (2 mL) were mixed with 400 μL of internal standard (undecanoic acid 1000 mg/L in hexane/acetone 80:20 *v*/*v*) and 2 mL of methanol/sulfuric acid (93:7 *v*/*v*). These mixtures were maintained for 60 min at 80 °C. Then, the samples were allowed to cool down to room temperature. After addition of 5 mL of hexane, the samples were mixed in a vortex for 1 min and two phases were formed. The organic phase was transferred to a volumetric flask (10 mL), and the re-extraction of the polar phase was carried out three to four more times until a volume of 10 mL was obtained. 

The FAMEs were analyzed with an Agilent 6890N GC (Agilent Technologies, Santa Clara, CA, USA), equipped with a SP2380 capillary column (30 m, 0.25 mm i.d., 0.20 μm film thickness) and flame ionization 19244–80560 as a detector. Nitrogen gas was used as carrier at a flow rate of 0.8 mL/min. The column temperature was kept initially at 50 °C for 2 min, then gradually increased to 240 °C at 4 °C/min, and finally, maintained at 240 °C for 1 min. An aliquot of the extracts (1 μL) was injected automatically with 20:1 in split mode. Injector and detector temperatures were set at 260 and 280 °C, respectively.

## 4. Conclusions

*Arthrospira platensis* showed a significant amount of carotenoids, tocopherols and fatty acids compared to other cyanobacteria species. These extracts represent a sustainable and safe source of functional lipophilic compounds. In general, SFE extractions with ethanol produced higher amounts of these compounds as compared with MAE extractions. This represents an important advantage because ethanol is an economical and environmentally friendly solvent. However, for carotenoids, MAE was more effective than SFE. The SFE extraction (BDF) with 450 bar, 60 °C and 53.33% of co-solvent gives the best extracts in terms of yield, tocopherols, carotenoids and fatty acids. For MAE, the extraction at 400 W and 50 °C (G) gives the best extracts in terms of tocopherols and carotenoids. For yield and fatty acids, the best extracts were prepared at 400 W and 70 °C (H). SFE and MAE proved to be suitable green extraction procedures for functional lipophilic compounds from *A. platensis*.

## Figures and Tables

**Table 1:** Experimental matrix design for conditions for supercritical fluid extraction (SFE) and microwave-assisted extraction (MAE), and extraction yields of *Arthrospira platensis* extracts.

Experiment *	P (bar)	T (°C)	CS (% of CO_2_)	Yield (% *w*/*w*) ^1^
ADE	150	60	26.70	3.09 ± 0.09 ^a,c,d^
BDF	450	60	53.33	4.07 ± 0.14 ^b^
ACF	150	45	53.33	3.05 ± 0.05 ^a,c,d^
ADF	150	60	53.33	3.13 ± 0.10 ^a,c.d^
ACE	150	45	26.70	1.21 ± 0.08 ^e^
BCE	450	45	26.70	1.40 ± 0.15 ^f^
BDE	450	60	26.70	1.96 ± 0.16 ^g^
BCF	450	45	53.33	1.72 ± 0.08 ^h^
G	1	50	–	2.03 ± 0.13 ^j^
H	1	70	–	4.27 ± 0.10 ^k^

* The extraction time for SFE and MAE was 50 and 15 min, respectively. Letters in the Experiment column are the acronym of the two levels of the tested variables: For SFE, Pressure (P) (150 (A), 450 (B)), Temperature (T) (45 (C), 60 (D)) and co-solvent (CS) (26.70 (E), 53.33 (F)). For MAE, Temperature (T) (50 (G), 70 (H)). ^1^ Values are represented as a mean ± standard deviation (*n* = 2 for SFE and *n* = 3 for MAE). ^a–h,j,k^ Different letters in the Yield column are significantly different (Least Significant Difference LSD test, *p* < 0.05).

## Supplementary Materials

Supplementary materials can be found at http://www.mdpi.com/1422-0067/17/5/658/s1.

## Abbreviations

SFE	Supercritical fluid extraction
MAE	Microwave assisted extraction
P	Pressure
T	Temperature
CS	Co-solvent
