# Supplementary material for: Supercritical Carbon Dioxide and Microwave-Assisted Extraction of Functional Lipophilic Compounds from Arthrospira platensis
Source: Int J Mol Sci. 2016 May 5;17(5):658. doi: 10.3390/ijms17050658 (PMC4881484; doi:10.3390/ijms17050658)
Supplement: Supplementary file 1 [file ijms-17-00658-s001.pdf]

Supplementary Material: Supercritical Carbon Dioxide and Microwave-Assisted Extraction of Functional Lipophilic Compounds from *Arthrospira platensis*

Diego A. Esquivel-Hernández, Víctor H. López, José Rodríguez-Rodríguez, Gibrán S. Alemán-Nava, Sara P. Cuéllar-Bermúdez, Magdalena Rostro-Alanis and Roberto Parra-Saldívar

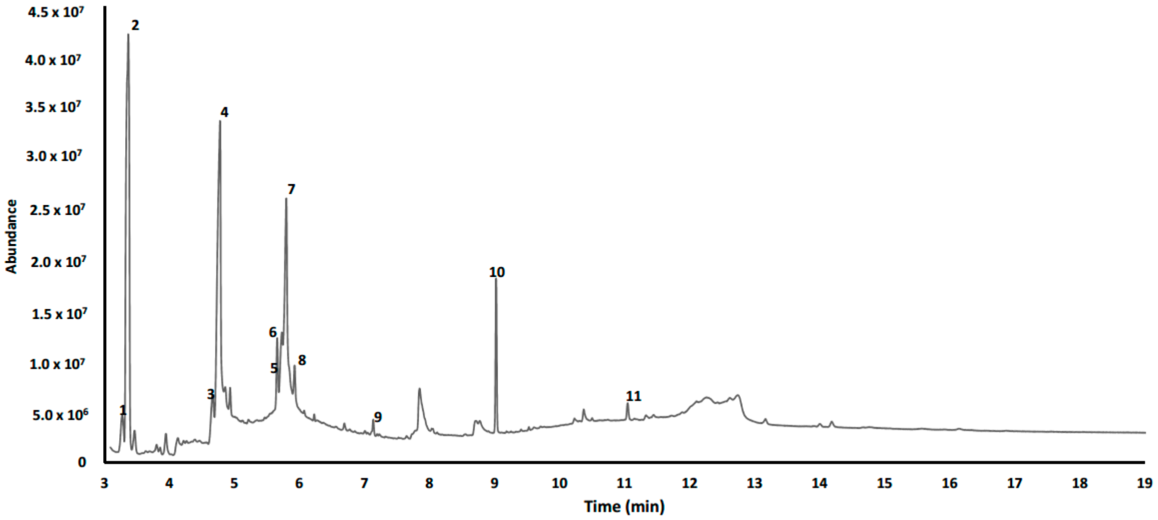

Figure S1. GC-MS chromatogram of volatile compounds present in ADF experiment from *A. platensis.* Note: The numbers corresponded to identified peaks* in Table S1.

Table S1. Volatile compounds present in ADF experiment from *A. platensis*.

Peak *	Rt (min)	Compound	Quality	Area (%)
1	3.277	8-Heptadecene	98	2.367
2	3.366	Heptadecane	99	29.485
3	4.666	Palmitoleic acid	99	3.474
4	4.778	n-Hexadecanoic acid	99	23.382
5	5.657	Phytol	98	2.501
6	5.728	1,9-Cyclohexadecadiene	96	4.496
7	5.799	9,12-Octadecadienoic acid (Z,Z)-	99	15.135
8	5.924	9,12-Octadecadienoic acid (Z,Z)-	98	2.432
9	7.135	9,12-Octadecadienoic acid (Z,Z)-	90	0.512
10	9.022	1,3-Benzenedicarboxylic acid, bis(2-ethylhexyl) ester	94	4.449
11	11.046	dl-α-Tocopherol	99	1.661

* Numbers corresponding to peaks in GC-MS chromatogram presented in Figure S1.
